# Brimonidine reduces TGF-beta-induced extracellular matrix synthesis in human Tenon’s fibroblasts

**DOI:** 10.1186/s12886-015-0045-8

**Published:** 2015-05-28

**Authors:** Samin Hong, Sueng-Han Han, Chan Yun Kim, Kang Yoon Kim, Yoo Kyung Song, Gong Je Seong

**Affiliations:** Institute of Vision Research, Department of Ophthalmology, Yonsei University College of Medicine, 50 Yonsei-ro, Seodaemun-gu, Seoul 120-752 Republic of Korea

**Keywords:** Fibroblast, Fibrosis, Brimonidine, Transforming growth factor-β, Glaucoma

## Abstract

**Background:**

Brimonidine is a highly selective α_2_ adrenergic agonist that has been widely used in anti-glaucoma eyedrops. The aim of this study was to investigate its putative anti-fibrotic role in the fibrosis caused by activated Tenon’s fibroblasts.

**Methods:**

Primary cultured human Tenon’s fibroblasts were exposed to 2.0 ng/mL of transforming growth factor-β1 (TGF-β1) for up to 48 h. In the presence of various concentrations of brimonidine (from 0.0 to 10.0 μM), the expression levels of fibronectin, collagen types I and III, and β-actin were determined by Western immunoblots. The expression of phosphorylated SMAD2/3 (p-SMAD2/3) was then evaluated using immunofluorescence.

**Results:**

TGF-β1 significantly increased the synthesis of fibronectin and collagens in human Tenon’s fibroblasts; however brimonidine treatment distinctly attenuated the TGF-β1-induced production of extracellular matrix (ECM) proteins. TGF-β1 also changed the cellular morphology to be plump, while brimonidine treatment returned the cells to a spindle shape, similar to control fibroblasts. Regarding p-SMAD2/3, brimonidine treatment did not show any apparent changes in its expression.

**Conclusions:**

Our data revealed that brimonidine reduces TGF-β-induced ECM synthesis in human Tenon’s fibroblasts *in vitro*. This finding implies that topical administration of brimonidine may be helpful in reducing the fibrosis caused by the long-term use of topical anti-glaucoma medications.

**Electronic supplementary material:**

The online version of this article (doi:10.1186/s12886-015-0045-8) contains supplementary material, which is available to authorized users.

## Background

Excessive subconjunctival fibrosis causes failure after glaucoma filtering surgery and transforming growth factor-β (TGF-β) is known to play a crucial role in this pathologic process. Though many anti-glaucoma eyedrops have been reported to induce conjunctival and/or subconjunctival fibrosis [1–3], information about the effects of brimonidine in this process remains scarce.

Brimonidine is a highly selective α_2_ adrenergic agonist that has been widely used as an anti-glaucoma ophthalmic solution [4–7]. It reduces intraocular pressure (IOP) by decreasing production of aqueous humor and increasing uveoscleral outflow. Several *in vivo* as well as *in vitro* studies have reported that brimonidine may directly protect retinal ganglion cells and optic nerve fibers in addition to lowering IOP; however a definite conclusion has not been made regarding its neuroprotective effects in human glaucoma patients.

Some previous reports dealt with the adverse effects of perioperative use of brimonidine for laser *in situ* keratomileusis (LASIK) [8–10]. Topical administration of brimonidine was found to raise the incidence of corneal flap dislocation after LASIK and Walter and Gilbert [8] suggested the following possible mechanisms: (1) brimonidine itself or brimonidine-containing eyedrops may act as a lubricant and cause the flap to slide from the corneal bed, (2) brimonidine may cause vasoconstriction of the anterior ocular vessels and decrease the corneal endothelial function to maintain proper flap adherence, and (3) brimonidine itself may be directly toxic to the corneal endothelial cells and reduce their metabolic activity. However, the precise mechanisms of this phenomenon have not yet been proven.

We hypothesized that brimonidine disturbs the healing/fibrotic process after LASIK surgery. Given than corneal fibroblasts, also known as keratocytes, are similar to Tenon’s fibroblasts, in the present study we attempted to assess whether brimonidine reduces TGF-β-induced extracellular matrix (ECM) synthesis in primary cultured human Tenon’s fibroblasts.

## Methods

### Cell culture and exposure to TGF-β1

Our protocol was approved by the Institutional Review Board of Gangnam Severance Hospital, Yonsei University College of Medicine, and all experiments were performed in compliance with the tenets of the Declaration of Helsinki. Subjects who had no ocular/systemic disease except for horizontal strabismus received comprehensive information and provided written informed consent. Patients with previous ocular surgery and/or trauma history were not included in the study.

Small Tenon’s capsule specimens were excised during strabismus surgeries and fibroblasts were isolated as previously described [11–13]. Cells were incubated in Dulbecco’s modified Eagle’s medium (DMEM; Life Technologies, Carlsbad, CA) supplemented with 10 % fetal bovine serum (FBS; Life Technologies), 100 units/mL penicillin (Life Technologies), and 100 μg/mL streptomycin (Life Technologies) at 37 °C and 5 % CO_2_. We used cells between the third and fifth passages for this study, and cultures were allowed to reach about 80 % confluence.

After 24 h of serum deprivation in serum-free DMEM, the fibroblasts were exposed to 2.0 ng/mL recombinant human TGF-β1 (R&D Systems, Minneapolis, MN) for up to 48 h. In the brimonidine treatment group, the cells were treated with various concentrations of brimonidine (Sigma-Aldrich, St. Louis, MO). All experiments were performed in at least quadruplicate and were repeated at least four times using independent cell cultures.

### Western immunoblots

Whole cellular proteins were extracted from primary cultured human Tenon’s fibroblasts. Briefly, total cell lysates were obtained using cell lysis buffer (Sigma-Aldrich) on ice for 10 min. The lysates were sonicated and the cell homogenates were centrifuged at 15,000 g for 10 min at 4 °C.

Protein concentrations in the resultant supernatants were determined with the Bio-Rad Protein Assay (Bio-Rad Laboratories, Hercules, CA) based on the Bradford dye-binding procedure. Equal amounts of protein (10 μg) were boiled in Laemmli sample buffer and resolved by sodium dodecyl sulfate polyacrylamide gel electrophoresis (SDS-PAGE). The proteins were transferred to polyvinylidene fluoride (PVDF) membranes and probed overnight with primary antibodies against human fibronectin, collagen types I and III, and β-actin (diluted 1:500; Santa Cruz Biotechnology, Dallas, TX).

Immunoreactive bands were detected with horseradish peroxidase-conjugated secondary antibodies (diluted 1:2,000; Santa Cruz Biotechnology) and visualized by an enhanced chemiluminescent system (SuperSignal West Pico Chemiluminescent Substrates, Pierce Biotechnology, Rockford, IL) on autoradiograph films.

### Immunofluorescence

The cells were fixed with 4 % paraformaldehyde for 30 min, treated with 0.1 % Triton X-100 in 0.1 % Na-Citrate for 10 min, and then blocked with 2 % bovine serum albumin (Sigma-Aldrich) for an hour. They were incubated with anti-human SMAD2/3 and phosphorylated SMAD2/3 (p-SMAD2/3) antibodies (1:50 dilution; Santa Cruz Biotechnology) overnight at 4 °C and then exposed to the Alexa Fluor® 594-conjugated and fluorescein isothiocyanate (FITC)-conjugated secondary antibodies (1:100 dilution; Life Technologies), respectively, for 60 min at room temperature. Mounting medium containing 4’,6-diamidino-2-phenylindole (DAPI; Santa Cruz Biotechnology) was applied and four random fields were imaged under a fluorescence microscope. SMAD2/3 was finally noticed with Alexa Fluor® 594 of red fluorescence, p-SMAD2/3 was labeled with FITC of green fluorescence, and all nuclei were counterstained with DAPI of blue fluorescence.

### Statistical analysis

Data are expressed as means ± standard error of the mean (S.E.M.). Data were compared using the Kruskal-Wallis one-way analysis of variance using the MedCalc program for Windows, version 11.4.2.0 (MedCalc Software bvba, Mariakerke, Belgium). P-values less than 0.05 were considered statistically significant.

## Results

**Fig. 1:**
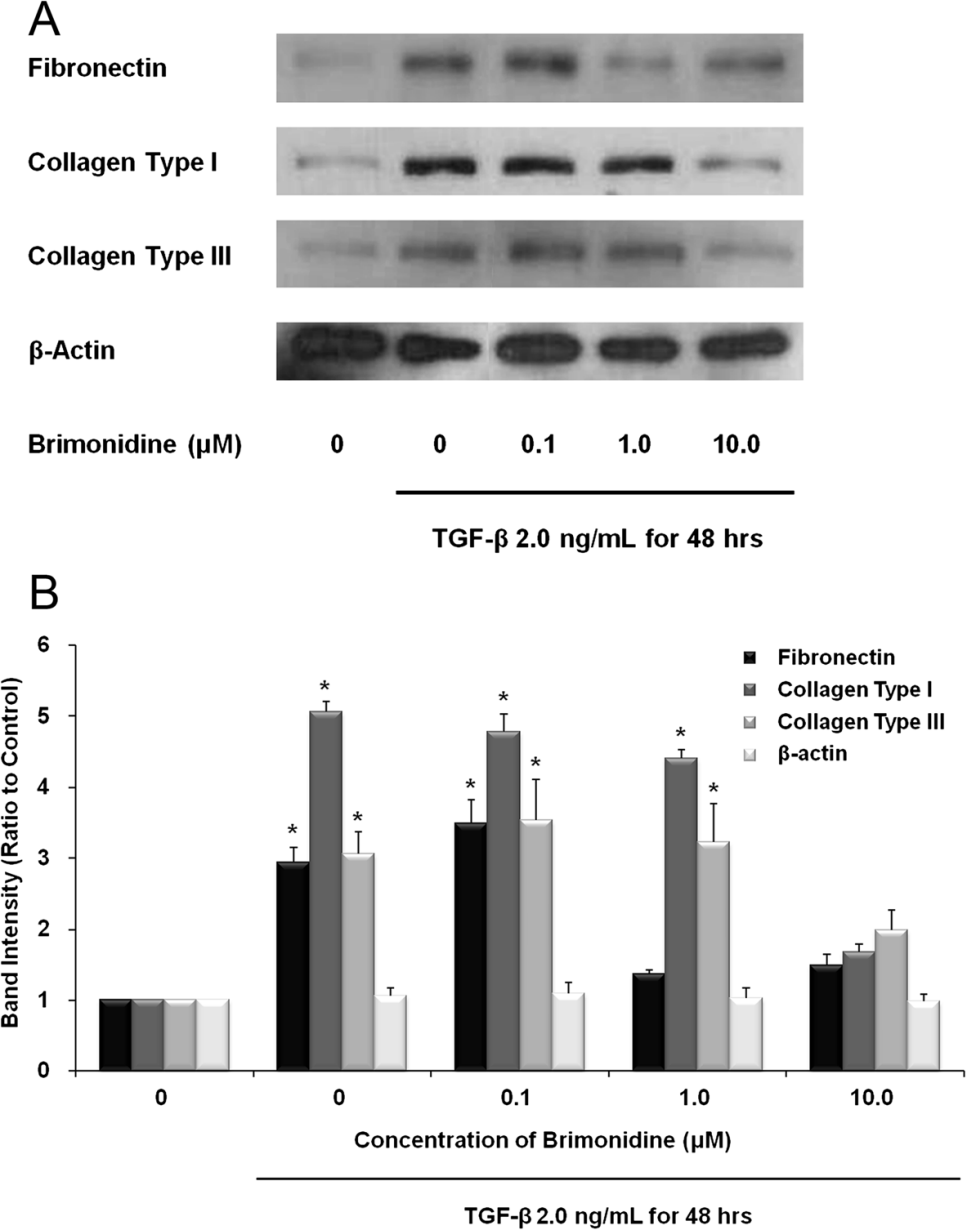
**a** Western immunoblots of primary cultured human Tenon’s fibroblasts. The cells were exposed to TGF-β1 (2.0 ng/mL) for up to 48 h in the presence of brimonidine. **b** Band intensity is expressed as ratio to control. Asterisk, p < 0.05

Data from the Western immunoblots are shown in Fig. 1. When human Tenon’s fibroblasts were exposed to TGF-β1 for 48 h, the levels of synthesis of fibronectin and collagen types I and III significantly increased. Brimonidine treatment distinctly attenuated this TGF-β-induced ECM protein synthesis. The anti-fibrotic effect of brimonidine on fibronectin was notable at a low concentration (1.0 μM) of brimonidine, while its effect on collagens was obvious at a high concentration of 10.0 μM.

**Fig. 2:**
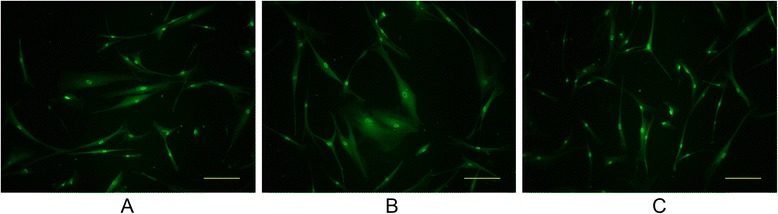
Representative immunofluorescence of primary cultured human Tenon’s fibroblasts. The cells were exposed to TGF-β1 (2.0 ng/mL) for 24 h in the presence of 10.0 μM brimonidine. **a** No treatment control; **b** TGF-β1 exposure only; **c** TGF-β1 exposure with brimonidine treatment. Phosphorylated SMAD2/3 was labeled with green fluorescence of fluorescein isothiocyanate. Scale bar, 200 μm

Representative immunofluorescence pictures are shown in Fig. 2. Human Tenon’s fibroblasts normally have a spindle shape (Fig. 2a). When the cells were exposed to TGF-β1 for 24 h, their cellular morphology became plump (Fig. 2b). Brimonidine treatment returned the cell shape to something similar to that of unstressed control cells (Fig. 2c). Furthermore, brimonidine treatment did not cause any apparent changes in the expression of p-SMAD2/3.

## Discussion

In this *in vitro* study, in order to investigate the anti-fibrotic effects of brimonidine on activated human Tenon’s fibroblasts, the primary cultured human Tenon’s fibroblasts were treated with 2.0 ng/mL of TGF-β1 for up to 48 h in the presence of various concentrations of brimonidine. As seen in the results of Western immunoblots, TGF-β1 significantly increased the protein synthesis of fibronectin and collagen types I and III compared to unstressed control cells. However, brimonidine treatment attenuated this TGF-β-induced increase in ECM protein synthesis.

Leng et al. [3] reported that human Tenon’s fibroblasts isolated from glaucoma patients with long-term use of topical anti-glaucoma eyedrops were highly proliferative and had elevated TGF-β expression compared to cells isolated from control subjects. Several earlier studies also demonstrated that the anti-glaucoma medications changed the expression levels of matrix metalloproteinases (MMPs) and tissue inhibitors of metalloproteinases (TIMPs), as well as the synthesis of interleukin (IL)-6 in Tenon’s fibroblasts cultured from glaucoma patients [14–16]. Though IL-6 is an essential chemoattractant and stimulator of lymphocytes [17], it participates in the core fibrotic process of transdifferentiation from Tenon’s capsule fibroblasts to myofibroblasts [11, 15]. Thus, all previous studies have produced a common idea that topical anti-glaucoma eyedrops may affect the fibrosis in glaucoma patients and influence the success of glaucoma filtering surgery. However, the previous studies are mainly regarding prostaglandin analogues rather than brimonidine. That is why we investigated the putative anti-fibrotic effect of brimonidine on human Tenon’s fibroblasts.

While it is not clear how brimonidine suppresses the TGF-β-induced ECM production in the cells, according to our immunofluorescence results, brimonidine does not seem to influence the activity of SMAD2/3, which are the receptor-regulated transcription factors that initiate the intracellular TGF-β signaling. Alpha adrenergic system is involved in tissue fibrosis and remodeling via modulation of the TGF-β signaling pathway [18]. Brimonidine may play its anti-fibrotic role via similar molecular cross-talk. To confirm the anti-fibrotic effects of brimonidine and to determine its precise mechanism, further *in vivo* as well as *in vitro* experiments are needed and are currently underway. In the future, the effects of brimonidine could also be confirmed by a human clinical trial. While it is not clear how brimonidine suppresses the TGF-β-induced ECM production in the cells, according to our immunofluorescence results, brimonidine does not seem to influence the activity of SMAD2/3, which are the receptor-regulated transcription factors that initiate the intracellular TGF-β signaling. It also does not have any significant effect on TGF-β-induced transdifferentiation of Tenon’s fibroblasts to myofibroblasts (Additional file 1). Alpha adrenergic system is involved in tissue fibrosis and remodeling via modulation of the TGF-β signaling pathway [18]. Brimonidine may play its anti-fibrotic role via similar molecular cross-talk. To confirm the anti-fibrotic effects of brimonidine and to determine its precise mechanism, further in vivo as well as in vitro experiments are needed and are currently underway. In the future, the effects of brimonidine could also be confirmed by a human clinical trial.

## Conclusion

Our data revealed that brimonidine reduces TGF-β1-induced ECM synthesis in human Tenon’s fibroblasts *in vitro*. These findings imply that brimonidine may be helpful in attenuating the fibrosis caused by the long-term use of topical anti-glaucoma medications.

## Additional file

Additional file 1: Representative Western immunoblots of primary cultured Tenon’s fibroblasts. The cells were exposed to TGF-β1 (2.0 ng/mL) for up to 48 hours in the presence of brimonidine. αSMA , α smooth muscle actin.

## Abbreviations

DAPI 4’: 6-diamidino-2-phenylindole
DMEM: Dulbecco’s modified Eagle’s medium
ECM: Extracellular matrix
FBS: Fetal bovine serum
FITC: Fluorescein isothiocyanate
IOP: Intraocular pressure
IL: Interleukin
LASIK: Laser *in situ* keratomileusis
MMP: Matrix metalloproteinase
PVDF: Polyvinylidene fluoride
p-SMAD2/3: Phosphorylated SMAD2/3
SDS-PAGE: Sodium dodecyl sulfate polyacrylamide gel electrophoresis
S.E.M.: Standard error of the mean
TGF-β: Transforming growth factor-β
TIMP: Tissue inhibitors of metalloproteinase
