# Supplementary figures and images for: Brimonidine reduces TGF-beta-induced extracellular matrix synthesis in human Tenon’s fibroblasts
Source: BMC Ophthalmol. 2015 May 28;15:54. doi: 10.1186/s12886-015-0045-8 (PMC4454273; doi:10.1186/s12886-015-0045-8)

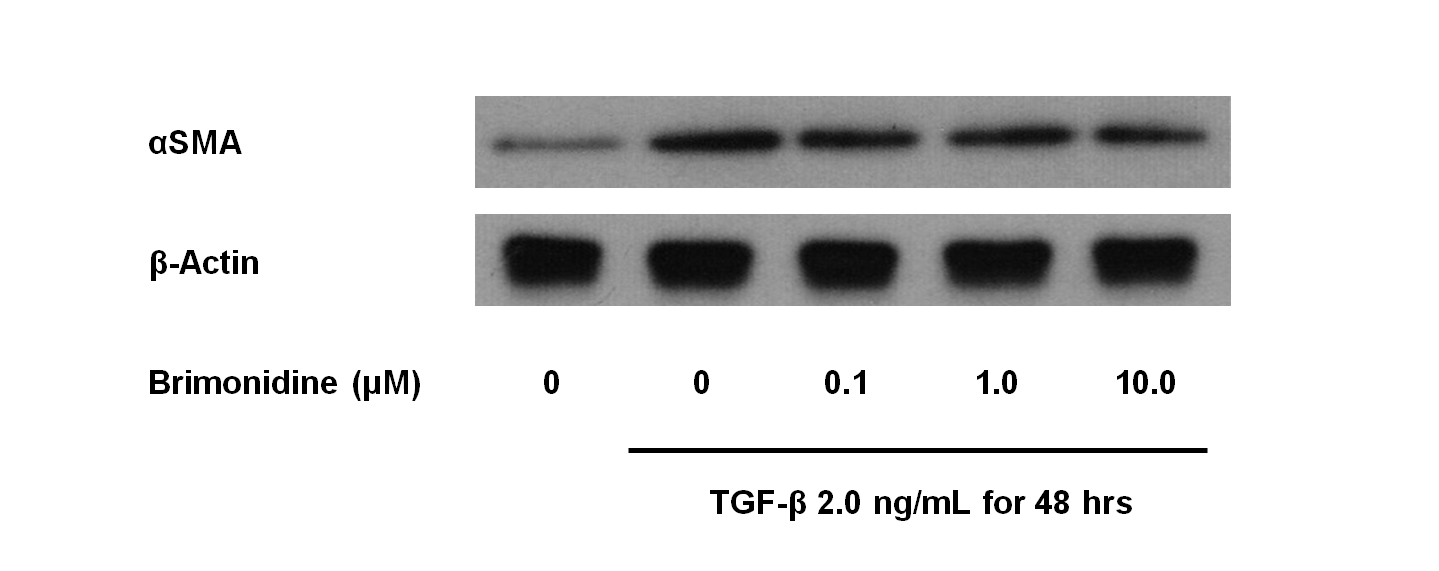

Supplement: Additional file 1: — Representative Western immunoblots of primary cultured Tenon’s fibroblasts. The cells were exposed to TGF-β1 (2.0 ng/mL) for up to 48 hours in the presence of brimonidine. αSMA , α smooth muscle actin. [file 12886_2015_45_MOESM1_ESM.tiff]
